# MicroRNA 399 as a potential integrator of photo-response, phosphate homeostasis, and sucrose signaling under long day condition

**DOI:** 10.1186/s12870-018-1460-9

**Published:** 2018-11-21

**Authors:** Lei Tian, Haiping Liu, Ligang Ren, Lixia Ku, Liuji Wu, Mingna Li, Shunxi Wang, Jinlong Zhou, Xiaoheng Song, Jun Zhang, Dandan Dou, Huafeng Liu, Guiliang Tang, Yanhui Chen

**Affiliations:** 1 College of Agronomy, National Key Laboratory of Wheat and Maize Crop Science, Henan Agricultural University, Zhengzhou, 450002 China; 2 Department of Biological Sciences, Michigan Technological University, Houghton, MI 49931 USA; 3 College of Life Science, Northwest Agriculture and Forestry University, Yangling, 712100 China; 4 Cereal Institute, Henan Academy of Agricultural Science/Henan Provincial Key Laboratory of Maize Biology, Zhengzhou, 450002 China

**Keywords:** microRNA399, Sucrose., Maize., *Arabidopsis thaliana.*, Photoperiod., Long day.

## Abstract

**Background:**

Photoperiod-sensitivity is a critical endogenous regulatory mechanism for plant growth and development under specific environmental conditions, while phosphate and sucrose signaling processes play key roles in cell growth and organ initiation. MicroRNA399 is phosphate-responsive, but, whether it has roles in other metabolic processes remains unknown.

**Results:**

MicroRNA399 was determined to be sucrose-responsive through a microRNA array assay. High levels of sucrose inhibited the accumulation of microRNA399 family under phosphate starvation conditions in *Arabidopsis thaliana*. Similarly, exogenous sucrose supplementation also reduced microRNA399 expression in maize at developmental transition stages. RNA sequencing of a near-isogenic line(photoperiod-sensitive) line and its recurrent parent Huangzao4, a photoperiod-insensitive line, was conducted at various developmental stages. Members of microRNA399 family were down-regulated under long-day conditions in the photoperiod-sensitive near-isogenic line that accumulated more sucrose in vivo compared with the control line Huangzao4.

**Conclusion:**

MicroRNA399s may play central roles in the integration of sucrose sensing and photoperiodic responses under long day conditions in maize.

**Electronic supplementary material:**

The online version of this article (10.1186/s12870-018-1460-9) contains supplementary material, which is available to authorized users.

## Background

Maize originated in southern Mexico in a tropical environment and required post-domestication selection to adapt to temperate ecological zones. Short-day-requiring tropical, but not temperate, maize shows a prevenient sensitivity response to a long photoperiod [1, 2]. Consequently, decreasing the sensitivity of photoperiodic regulation is an effective way to obtain new maize varieties. In *Arabidopsis thaliana*, numerous genes that are implicated in the control of the plant circadian clock and photoperiodicity have been extensively studied to dissect the molecular mechanism of flowering regulation [3–6]. In maize, however, only a small number of genes, such as *Zea mays CENTRORADIALIS1 (ZCN1)*, *ZCN8*, and as well as the CONSTANS-like gene *CONZL*, have been associated with the vegetative-to-reproductive phase transition and flowering time [7–9]. *ZmCCT,* which encodes a CCT domain-containing protein, is an important gene related to photoperiodic flowering [10]. Teosinte *ZmCCT* alleles are consistently expressed at higher level than temperate maize alleles under long day (LD) condition [11]. In our previous studies, by generating the populations from a cross between the temperate inbred line Huangzao4 (H4, the recurrent parent) and the tropical inbred line CML288 (the donor parent), we mapped qDPS10 to a 130-kb region and by the bioinformatics analysis. The CCT domain gene (*GRMZM2G381691*) in qDPS10 was considered as a candidate gene for photoperiod sensitivity. Additionally, higher transcript levels were observed in the *ZmCCT*-associated near-isogenic line (NIL), than that in its recurrent parent, H4 under LD conditions [12].

In addition, circadian rhythms are influenced by environmental signals, such as light and temperature, and by endogenous sugar production from photosynthesis to enable plants to adapt to local environments [13, 14]. Sucrose, the major type of sugar produced by photosynthesis in plants, is a pivotal energy and carbon resource for plants. Sucrose also acts as a central sensing and signaling molecule in plant metabolic pathways [15–19] and is the main form of sugar transported from mature leaf mesophyll cells to roots, stems, and other organs. High levels of endogenous sucrose repress photosynthesis and nutrient mobility [20, 21].

MicroRNAs (miRNAs) are endogenous, non-coding, single-stranded small RNAs approximately 21–24 nucleotides long. Many miRNAs regulate flowering time in various species by cleaving or repressing the translation of their target mRNAs [22–27]. In *A. thaliana*, miR156 and its target SQUAMOSA PROMOTER BINDING PROTEIN-LIKE (*SPL*) genes constitute an endogenous flowering pathway, and SUPRESSOR OF OVEREXPRESSION OF CONSTANS1 (*SOC1*) and FLOWERING LOCUS T (*FT*) can directly bind to the promoter regions of *SPL3*, *SPL4*, and *SPL5* and regulate them in a photoperiod-dependent manner [28]. In addition, GIGANTEA (*GI*)-mediated photoperiodic flowering in *A. thaliana* is coordinately regulated by two different genetic pathways: one mediated by CONSTANS (*CO*) and the other by miR172 and its targets [26]. MiR399 family members are phosphate-deficient responsive and involved in phosphate homeostasis [29–32]. The miR399s and sucrose are phloem-mobile molecules [31–34], and they may potential interact in the signaling pathway would be particularly interesting. In addition, little is known about the functions of miR399s in photoperiodic response and flowering time determination in maize.

In this study, we aimed to better understand sucrose-responsive miRNAs and the cross-talk that occurs during long photoperiods, miR399s, and sucrose homeostasis in *A. thaliana* and maize. We screened sucrose-responsive miRNAs using miRNA array technology in *A. thaliana*. Small RNA sequencing was also carried out to identify photoperiod-regulated miRNAs in photoperiod-insensitive inbred maize line H4 and its near-isogenic line NIL, the latter is sensitive to long photoperiods. The miR399s were fully repressed by high sucrose levels at earlier leaf developmental stages. The onset of miR399 expression was extremely sensitive to long photoperiods during the development of maize. In addition, the NIL accumulated a high sucrose level in vivo*,* which suggested that miR399s participate in photoperiod-regulated networks in maize in response to LD conditions by modulating sucrose accumulation.

## Methods

### Plant materials and growth conditions

*A. thaliana* ecotype Columbia plants were used in this study*. A. thaliana* seeds were collected in growth chambers (60% humidity, 100 μmol m^− 2^ s^−1^under LED light) in Houghton, Michigan, USA. These seeds were surface sterilized with 70% ethanol for 1 min and then soaked in 10% bleach for 20 min with agitation every 2 to 3 min. The sterilized seeds were rinsed at least five times with sterilized water and then sown under a hood on agar-solidified Murashige–Skoog (MS) salt medium. The petri-dishes with seeds were maintained at 4 °C for 72 h to homogenize germination and break dormancy. Seven days after germination, we transferred the seedlings at similar growth states to culture bottles containing MS medium supplemented with low (1%) or high (6%) level of sucrose or into a phosphate-deficient MS medium with low or high levels of sucrose. All of the seeds were then transferred to a controlled growth chamber set to 22 ± 1 °C with a 15-h light (100 μmol m^− 2^ s^− 1^)/9-h dark photoperiod, which represented LD conditions.

The photoperiod-sensitive NIL, was the cross product of inbred line H4 and the tropical maize inbred line CML288 described in our previous studies [12]. The latter was acquired from the National Maize and Wheat Improvement Center (CIMMYT), Mexico City, Mexico, while the former, which was the recurrent parent, was a representative of the Chinese inbred line Tangsipingtou. Four plants were grown per pot in 15-cm pots in liquid full-strength Hoagland’s medium in the dark in the growth chambers described previously [12].

Seedlings at the four-fully expanded leaf stage were used for the following experiments. Before the sucrose treatment, the seedlings were starved of sucrose by placing them in the dark for 24 h to completely starve them for sugar [35]. Sets of four-fully expanded leaf stage seedlings were then transferred to full-strength Hoagland’s medium containing either 0.25 mM KH_2_PO_4_ (P+, control) or no additional phosphate (P−,) in the dark. One set of the P+ and P− media contained 3% sucrose (S, LP + S), while another set was without sucrose (LP). Untreated samples (0.25 mM KH_2_PO_4_) were maintained as controls and were collected at the same time as the treated samples. Nutrient solutions were renewed daily to ensure pH stability. Each treatment was repeated for three times. Newly developed leaves from three plants per replicate were collected per 12 h, then frozen immediately in liquid nitrogen, and stored in a − 80 °C freezer until use. Three biological replicates were performed.

The maize and *Arabidposis* seeds used in this study were provided by Dr. Cuiling Wang (Henan University of Science and Technology), and Prof. Hairong Zhang (Henan Agricultural University), respectively.

### Starch and sucrose measurements

H4 and NIL plant material were selected and collected during four previously defined developmental periods from plants in growth chambers (2.8 × 5.6 × 8.2 m) under LD conditions (15-h light/9-h dark, 25 °C, 100 μmol m^−2^s ^− 1^ light intensity) [12]. We defined four developmental stages according to our previous study [12]. In photoperiod-insensitive inbred line H4, the vegetative developmental periods corresponded to the period that the SAM always differentiated into leaf primordium (V1: three-fully expanded leaf stage and V2: four-fully leaf stage), whereas reproductive periods that SAM shapes elongated and become a cone (V3: five-fully leaf stage and V4: six-fully leaf stage). In photoperiod-sensitive inbred line NIL, the vegetative developmental periods corresponded to the period that the SAM always differentiated into leaf primordium (V1: three-fully expanded leaf stage and V2: five-fully leaf stage), whereas reproductive periods that SAM shapes elongated and become a cone (V3: six-fully leaf stage and V4: seven-fully leaf stage).

The newly expanded leaves of H4 and NIL at the four leaf stages were separately ground to fine powders with liquid nitrogen using mortars and pestles. Subsequently, 200 mg of each powdered sample were briefly homogenized with 600 μl of distilled water in a microcentrifuge tube and then immediately heated in boiling water for 10 min. After centrifugation at 20,000×*g* for 10 min at 4 °C, 100 μl of the supernatant was assayed with a spectrophotometer (Hitachi U-2900; Hitachi, Waltham, MA, USA). A commercial assay kit was used to measure the starch content of the insoluble carbohydrate fraction following the manufacturer’s instructions (R-Bio-pharm, Darmstadt, Germany). A unique kit was used to determine the sucrose content of the soluble carbohydrate fraction according to the manufacturer’s instructions (K-SUFRG Megazyme, Bray, Ireland) as previously described [36]. Newly expanded leaves from five plants each of H4 and NIL were examined as one biological replicate, and the average values were based on three biological replicates.

### Maize RNA extraction, deep sequencing, and data analysis

For RNA sequencing(RNA-Seq), we collected leaves and SAM tissue from the same plant in growth chambers under LD conditions as described by our previous study [12]. For leaves, equal amounts (mixed from five seedlings) of samples from the mid part of the top fully-expanded leaves were collected at V1, V2, V3, and V4 stages from H4 and NIL plants, respectively. For SAM, we collected equal amounts stem tips (mixed from five seedlings) described by the morphology observations in our previous study [12]. At each stage, 14 uniform growth seedlings were harvested. The leaf and SAM samples were collected from the same set of five seedlings at each stage for each maize inbred line, respectively. Another nine seedlings (three seedlings per replicate) were combined to analyze gene expression using quantitative reverse transcription PCR (qRT-PCR). Three independent biological replicates were used for the gene expression validation. All of the samples were flash-frozen in liquid nitrogen and then stored at − 80 °C. Total RNA for deep sequencing was isolated using a Plant Total RNA Extraction kit (Bioteke Corporation, Beijing, China) and then treated with DNase I and magnetic oligo(dT) beads following the manufacturer’s protocol. cDNA was synthesized using random hexamers and SuperScript II Reverse Transcriptase (Life Technologies, Ontario, Canada). The cDNA libraries were sequenced with 100-bp paired-end reads on the HiSeq 2000 platform (Illumina, Beijing, China) at the Beijing Genomics Institute.

Low-quality reads were removed from the raw data, and the appropriate small RNAs were mapped to miRNAs reported in miRBase (http://www.mirbase.org/). To compare relative expression levels, sequence counts were normalized in terms of reads per million (RPM).

After normalization, miRNA sequences present at levels < 1 RPM in the four samples were discarded [37]. For the identification of novel miRNAs, the Mireap software program was used to analyze unannotated small RNA reads. Small RNAs fulfilling the strict criteria described by Ding et al. [38] were deemed to be novel miRNAs. Only those candidates with a minimal folding free energy index > 0.85 were treated as novel maize miRNAs [38].

The differential expression of miRNAs between two inbred lines was analysed by DESeq [39]. MiRNAs were considered up- or down-regulated on the basis of two criteria: a |log2 fold change| > 1 and a false discovery < 0.05, and *P*-value could be assigned to each miRNA and adjusted by the Benjamini and Hochberg’s approach for controlling the false discovery rate.

### Computational predictions and functional analyses of maize miRNA targets

The potential targets of miRNAs in maize were predicted using psRNATarget software with default parameters [40]. The target searching was performed using the maize PlantGDB genomic CD library. Potential miRNA targets were functionally annotated by comparing them with the gene ontology (GO) database using AgriGO (http://bioinfo.cau.edu.cn/agriGO/) with default parameters.

### qRT-PCR validation in maize

To validate the miRNA expression levels identified by RNA-Seq, we performed the qRT-PCR analysis of all differentially expressed miRNAs in this study. Total RNAs from the fully expanded leaves and SAM of six H4 and NIL plants harvested at each of the V1, V2, V3, and V4 stages were extracted using RNAiso Plus (Takara, Dalian, China). Three biological replicates were performed. cDNA was synthesized using a miRNA cDNA synthesis kit (Takara) following the manufacturer’s protocol. The reverse transcription of small RNAs was carried out using a SYBR Prime Script miRNA RT-PCR kit (Takara) according to the manufacturer’s instructions. Validation of miRNAs by qRT-PCR was performed on a CFX9 Real-Time System (Bio-Rad, Hercules, CA, USA) using SYBR Premix Ex *Taq* II (Takara) and Roche FastStart Universal SYBR Green Master (Rox) mix (Roche, Basel, Switzerland), respectively. The 2^–ΔΔCt^ relative quantification method was used to analyze relative gene expression levels, using the 5S RNA (miRNA qRT-PCR) and 18S RNA (target gene qRT-PCR) genes used as standards. To verify the sequencing results, 10 conserved and 5 novel miRNAs were selected for amplification. Primer specificity was verified by a melting curve analysis. The reverse primer used for qRT-PCR amplification of miRNAs was the Uni-miR qRT-PCR primer in the miRNA cDNA synthesis kit. The remaining qRT-PCR primers are listed in the Additional file 1. Three biological replicates in technical triplicate were performed for all samples. Data were statistically analyzed using ANOVA, and means were compared using Duncan’s multiple range test at *P* < 0.05 level in SPSS V. 20.0.

### *A. thaliana* RNA isolation and northern blot analysis

Total leaves RNAs from *A. thaliana* grown in MS salt medium supplemented with 1% or 6% sucrose for 3 weeks and then in a phosphate-free medium for 2 weeks were isolated using Trizol reagent (Takara). The protocol for the northern blot analysis was previously described [41]. Briefly, a 20-μg aliquot of each RNA sample was mixed with RNA sample buffer and then separated on a 15% (*w*/*v*) urea-PAGE denatured gel. The separated RNAs were electro-transferred to Hybond-N membranes (Amersham Biosciences, Beijing, China), which were then rinsed with 2× SSPE buffer to remove gel fragments. RNAs were further fixed to membranes by UV cross-linking. After pre-hybridization for 2 h in hybridization buffer at 37 °C, hybridization was performed in hybridization buffer containing DNA oligonucleotides labelled with [γ-P^32^] ATP overnight at 37 °C. After hybridization, membranes were washed with 2× SSC/0.1% (w/v) SDS at 37 °C for 30 min; this process was repeated three times. Autoradiography was carried out by maintaining blotted membranes overnight on phosphorimager screens, followed by scanning with a Typhoon scanner. ImageQuant software was used for quantification of the radio signals.

## Results

### Growth traits and sucrose contents in photoperiod-sensitive and photoperiod-insensitive maize inbred lines under LD conditions

**Fig. 1:**
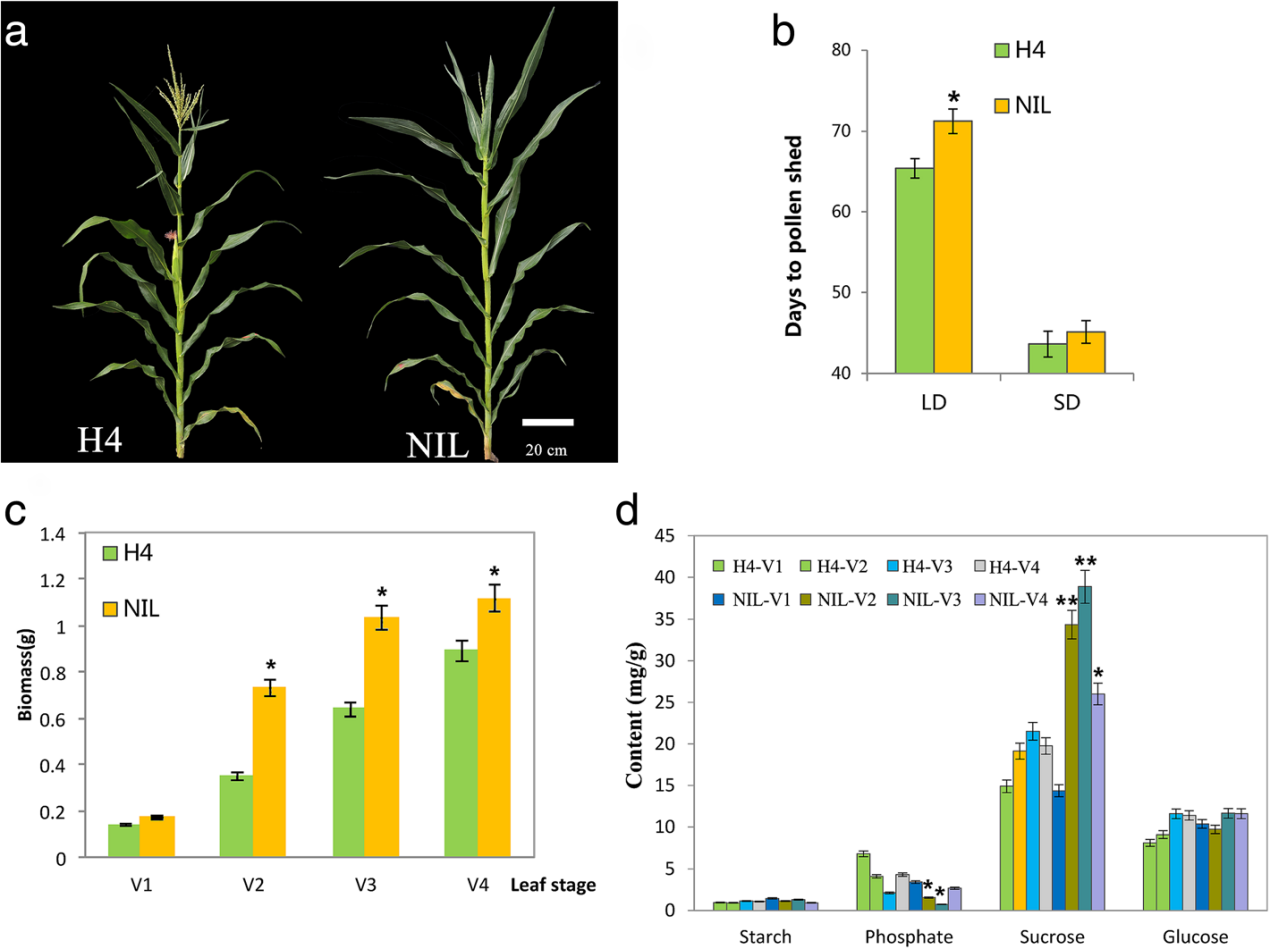
Phenotypes and growth parameters of maize lines H4 and NIL. **a** Plant morphology of maize inbred line H4 and its near isogenic line NIL under long-day conditions. **b** Days to pollen shed under long-day (Zhengzhou, Henan) and short-day (Sanya, Hainan) conditions. **c** Biomasses of maize seedlings of the inbred H4 and NIL at different developmental stages (means ± SEs, *n* = 6). **d** Starch and sucrose contents of H4 and NIL seedlings. Significant differences between H4 and NIL were assessed using Student’s *t*-test; * *P* < 0.05, ** *P* < 0.01

Under LD conditions, NIL plants were considerably taller than those of the H4 line (Fig. 1a). H4 plants showed less photoperiodic sensitivity than NIL plants, which exhibited a one-week delay in flowering under LD conditions (Fig. 1a and b). To examine changes in the sucrose contents of maize seedlings in response to photoperiod, individual samples of H4 and NIL, were periodically collected and the plant phenotypes were observed at V1, V2, V3, and V4 stages under LD conditions. At the V2 stage under LD conditions, the NIL accumulated significantly greater biomass and sucrose content than H4, whereas relatively steady levels of starch and glucose were identified during these stages (Fig. 1c and d). Thus, a long photoperiod had an important influence on flowering time and the accumulation of sucrose in vivo.

### Identification of miRNA expression patterns in leaves and SAM under LD conditions during the four studied maize leaf stages

**Fig. 2:**
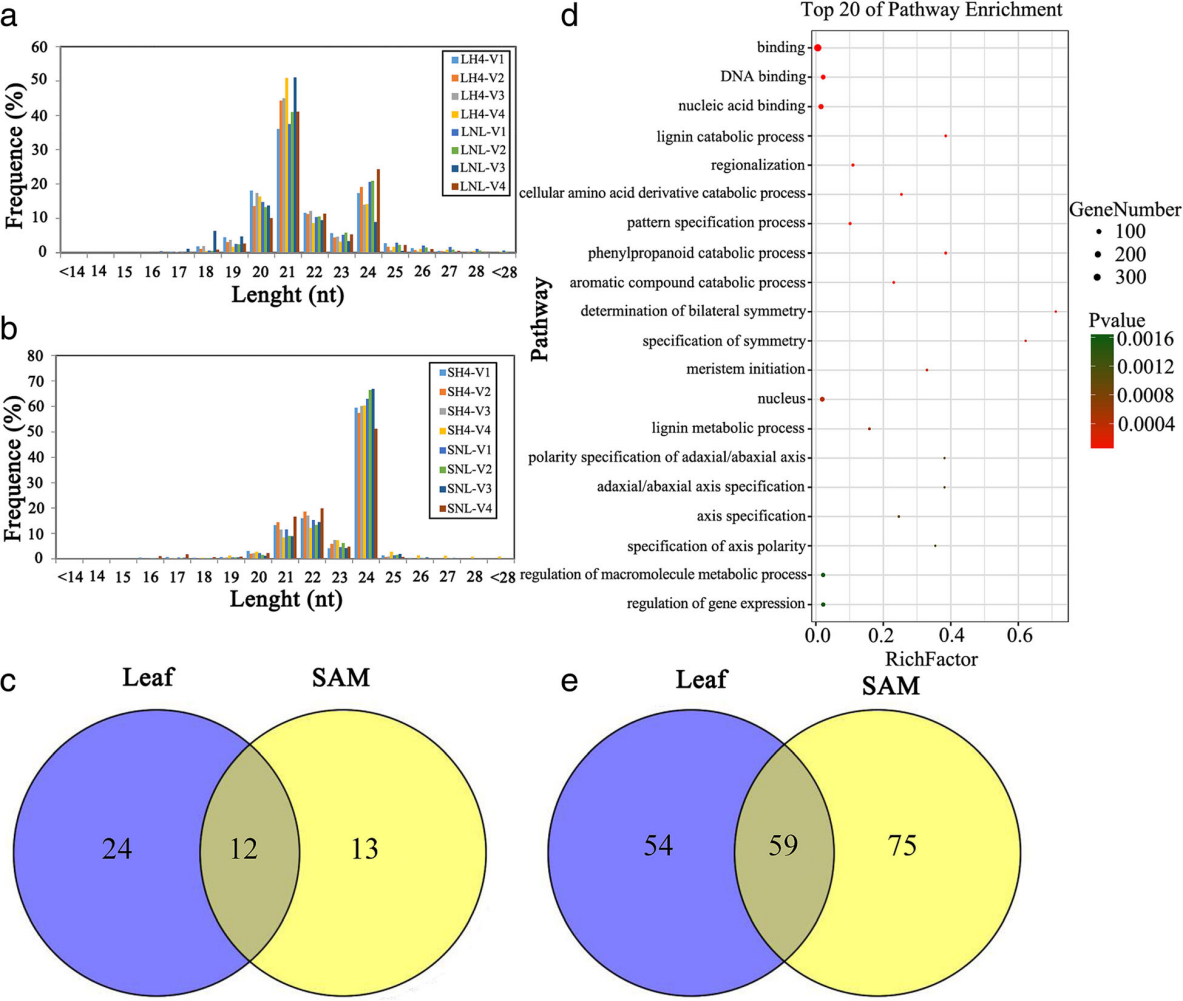
Characteristics of small RNA libraries from leaves and SAM of maize H4 and NIL obtained by miRNA sequencing. **a-b** Comparison of size distributions of small RNA libraries in leaves (**a**) and SAM (**b**) of H4 and NIL. (**c**) Venn diagram of novel miRNAs in leaves and SAM of H4 and NIL (**d**) Top 20 of GO functions significantly enriched with target genes of both conserved and novel predicted miRNAs obtained from RNA sequencing. e Venn diagram of differentially expressed conserved miRNAs in leaves and SAM between H4 and NIL

To investigate potential differences in miRNA expression patterns during photoperiod-dependent floral transitions between H4 and NIL plants under LD conditions, we performed small RNA-Seq using SAM tissues and leaves from the V1 to V4 stages of these two genotypes grown under LD conditions. This sequencing generated 13 to 23 million reads from each H4 or NIL sample. After removing adapters and low-quality reads, the remaining clean reads, representing 96% of the total reads, were retained for further analysis (Table 1). As shown in Table 1, more than 55% of these reads could be matched perfectly to the maize genome. Analysis of small-RNA length distributions (Fig. 2a and b) in the four maize samples indicated a peak size of 21 nt from leaves and 24 nt from SAM, which suggested the organ-specific accumulation of miRNAs.

**Table 1:** Summary of the miRNA sequencing of individual libraries

	total reads	clean reads	unique reads	perfectly mapped to genome
LH4-V1	17,186,960	16,963,404(98.69%)	2,052,631(12.10%)	1,197,905(58.35%)
LH4-V2	13,380,625	13,243,556(98.97%)	2,087,027(15.75%)	1,233,404(59.09%)
LH4-V3	17,751,014	17,568,846(98.97%)	2,374,513(13.51%)	1,358,280(57.20%)
LH4-V4	19,505,369	19,300,302(98.94%)	2,116,078(10.96%)	1,221,134(57.70%)
LNIL-V1	10,007,901	9,881,920(98.74%)	1,665,835(16.85%)	961,709(57.73%)
LNIL-V2	17,805,863	17,645,763(99.10%)	2,697,086(15.28%)	1,551,514(57.52%)
LNIL-V3	22,374,571	21,819,878(97.52%)	1,811,746(8.30%)	1,057,255(58.35%)
LNIL-V4	21,309,723	21,052,171(98.79%)	3,222,375(15.3%)	1,793,192(55.64%)
SH4-V1	22,807,477	22,338,639(98.45%)	6,313,116(28.26%)	3,650,905(57.83%)
SH4-V2	18,426,335	18,224,946(99.47%)	5,301,591(29.08%)	3,075,100(58.00%)
SH4-V3	20,899,478	20,677,910(99.53%)	6,382,101(30.86%)	3,724,696(58.36%)
SH4-V4	23,288,648	23,026,184(99.45%)	7,010,719(30.44%)	4,070,374(58.05%)
SNIL-V1	21,959,560	21,639,576(99.07%)	6,242,915(28.84%)	3,617,275(57.94%)
SNIL-V2	19,457,112	19,218,834(99.35%)	6,461,810(33.62%)	3,737,160(57.83%)
SNIL-V3	19,713,918	19,465,370(99.27%)	5,947,829(30.55%)	3,418,295(57.47%)
SNIL-V4	19,396,171	18,651,525(96.69%)	5,076,368(27.21%)	2,957,408(58.25%)

miRDeep2 was used to identify known miRNAs based on clean reads obtained from the RNA-Seq. In total, 197 known miRNAs belonging to 27 different families were identified in the two inbred lines (Additional file 2). The two largest conserved families were miR166 and miR399, with 22 and 18 members, respectively (Additional file 2). Twelve novel miRNA candidates were co-detected in leaves and SAM from the two inbred lines, and 24 miRNAs were specifically predicted in leaves, while 13 were identified in SAM (Fig. 2c). However, most of the novel predicted miRNAs were expressed at relatively low levels and were exclusively detected in one or two libraries.

### Prediction of miRNA targets in maize leaves and SAM

To predict targets of both conserved and novel predicted miRNAs obtained from RNA-Seq, we used psRNATarget, which revealed 1009 targets of 291 known miRNAs and 86 targets of 12 novel miRNAs (Additional file 3). Potential functional annotations of the miRNA targets were performed using GO analyses [42]. The 1095 target genes were assigned to 73 significant GO terms (*P* < 0.05) (Fig. 2d; Additional file 4). Binding (GO: 005488) was a dominant term in the category of molecular function, corresponding to 77.1% of 428 genes. Regionalization (GO: 0003002) was enriched in the biological process category (Fig. 2d). Floral-related functions of target genes were further identified, such as floral organ development (GO: 0048437), floral whorl development (GO: 0048438), and flower development (GO: 0009908) (Additional file 4).

### MiRNAs expression patterns in response to LD conditions in maize leaves and SAM

As revealed by the deep sequencing results, the expression levels of almost all miRNAs changed during the progression of maize development from the V1 to V4 stages. Totally, 113 and 134 known miRNAs displayed significantly different expression levels in maize leaves and SAM, respectively (Fig. 2e). As shown in Additional file 5, in contrast to other LD-induced miRNAs, miR399d and miR399j were strongly induced in H4 leaves by LD conditions, with a greater than 10-fold expression difference at the V2 and V3 stage. The expressions of miR399d, and miR399i were LD-induced in SAM but significantly down-regulated in NIL (Additional file 5). This result indicated that miR399 plays an important role in the LD photoperiodic regulation of both the NIL’s leaves and SAMs.

**Fig. 3:**
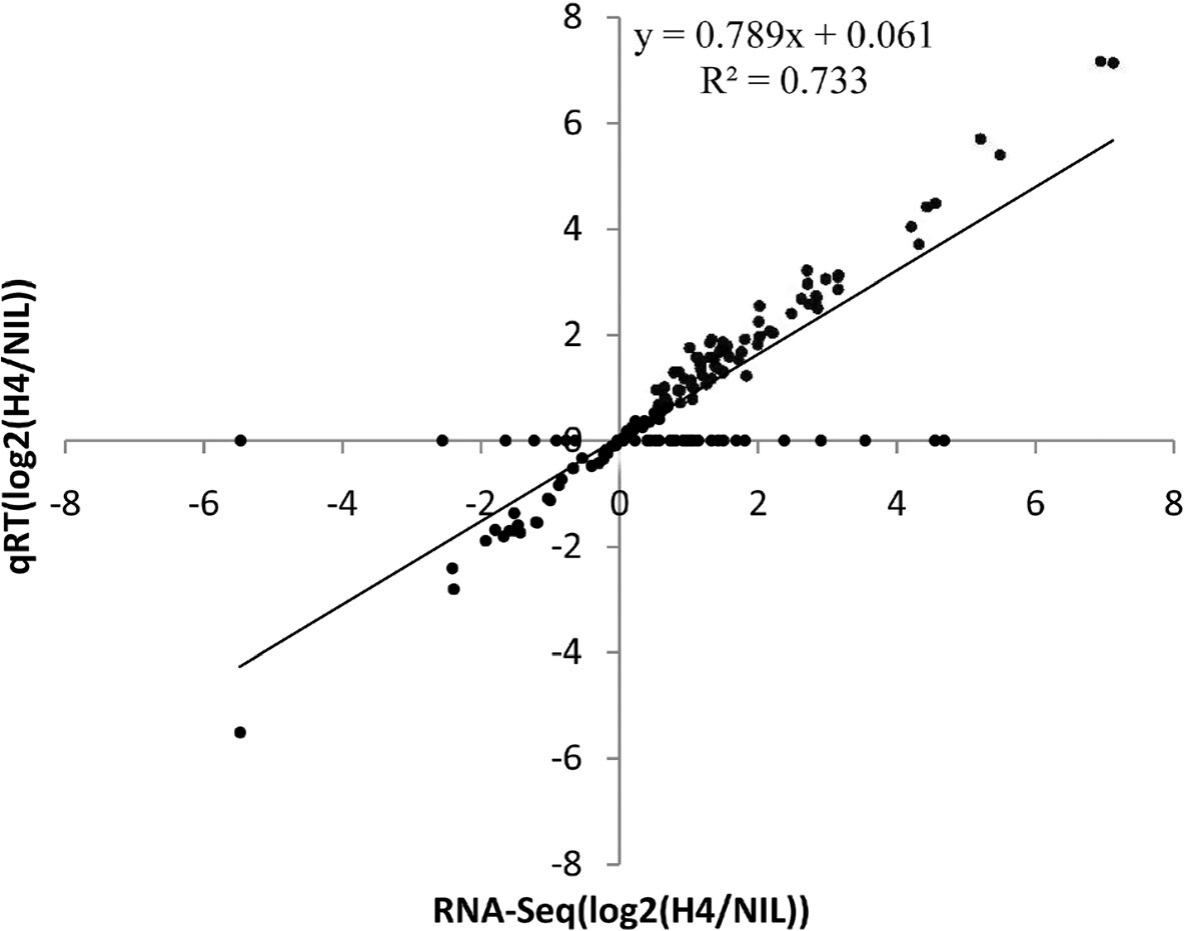
Validation of differentially expressed miRNAs inferred from RNA sequencing data between the maize H4 and NIL using quantitative real-time PCR. Seventeen miRNAs exhibiting diverse expression profiles in the RNA-seq data were selected for qPCR analysis. The average value of each gene based on RNA-seq expression data was plotted against that from qPCR result and fit into a linear regression. Both x- and y-axes are indicated by log2 scales

To validate the miRNAs identified by RNA-Seq in maize leaves and SAM, 5 novel maize miRNAs (miRn018, miRn023, miRn024, miRn027, and miRn032) and 10 conserved miRNA families (miR156, miR159, miR167, miR169 miR172, miR390, miR393, miR398, miR399 and miR827) were subjected to qRT-PCR analyses. Relative expressions of selected miRNAs in H4 and NIL during the four studied developmental stages as assessed by qRT-PCR were consistent with those from the deep-sequencing data, with a relative R^2^ value of 0.733 (Fig. 3).

### The effect of external sucrose on miR399 expression in maize

**Fig. 4:**
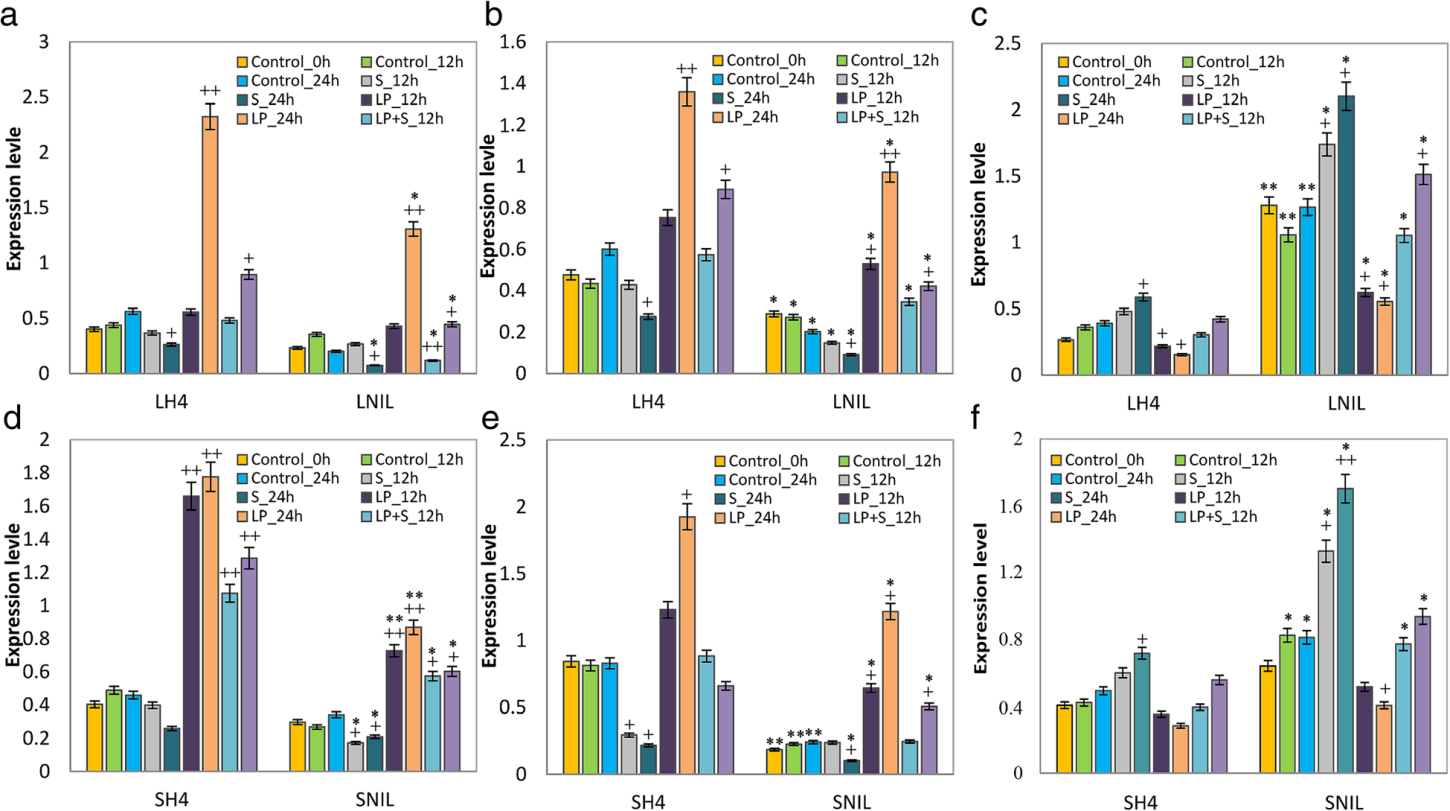
Relative expression levels of miR399a (**a** and **d**), miR399d (**b** and **e**), and *PHO2* (**c** and **f**) in leaves (upper panel) and SAM (lower panel) of maize subjected to external sucrose treatments. Sets of 4-fully expanded leaf stage seedlings were then transferred to the Hoagland’s medium containing either 0.25 mM phosphate (P+, Control) or no additional phosphate (P−, LP). One set of the P+ and P− media contained 3% sucrose (S, LP + S), while another set was not supplemented with sucrose (LP). Two days later, plants were harvested. Error bars indicate SDs (*n* = 3). Significant differences were evaluated using Student’s *t*-test; *^, +^ *P* < 0.05, **^, ++^ *P* < 0.01. ^+^ refers to significant differences between each treatment and the control; *, indicates significant differences between the maize H4 and NIL

The sharp expressional changes of miR399s during the transition from vegetative to reproductive phases indicated that this miRNA family is associated with flowering time under LD conditions in maize. In addition, more sucrose accumulated in the NIL than in H4 in response to LD condition. To explore the relationships between miR399s and sucrose accumulation in maize, H4 and NIL seedlings were subjected to high sucrose and phosphate deficiency. MiR399a and miR399d showed significantly elevated expression levels in both H4 and NIL under LD plus phosphate starvation conditions; however, the inbred lines grown under phosphate deficiency and in the presence of sucrose (P− S+) had relatively lower miR399 expressions levels than those grown in the absence of both compounds (P− S−). In addition, seedlings subjected to high sucrose exhibited lower miR399 expression levels compared with those grown under other treatment conditions (Fig. 4). In contrast, the target genes of the miR399s displayed opposite expression patterns compared with those of the miR399s under these treatment conditions. Thus, external sucrose supplementation may inhibit miR399 expression in maize. The expression of miR399 was lower in the NIL than in H4, which further supported our previous conclusions based on RNA-Seq results from these two inbred lines.

### Temporal expression of phosphate homeostasis-related genes under long-day conditions in maize

**Fig. 5:**
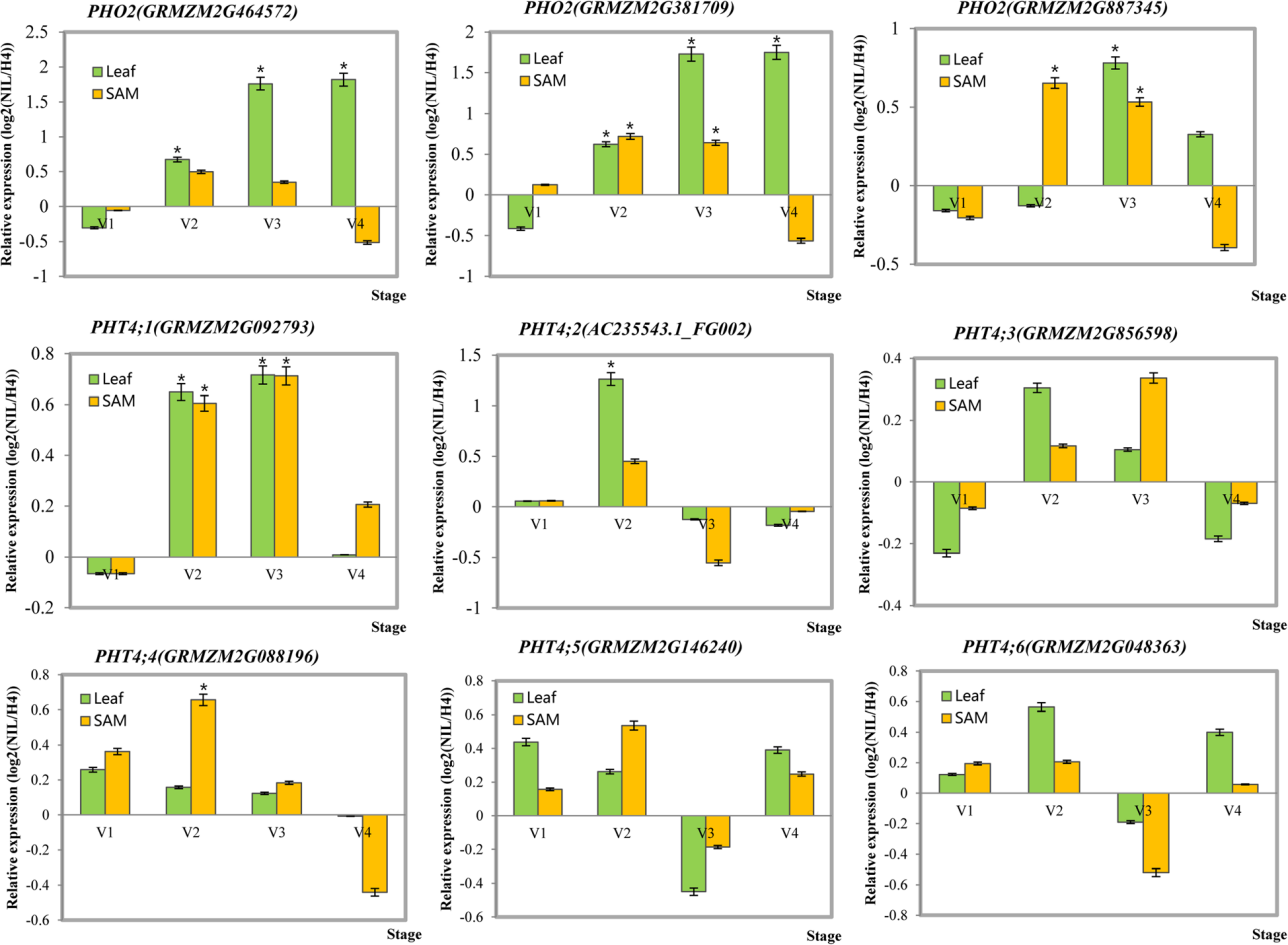
Expression levels of genes downstream of the miR399s in the maize H4 and NIL lines. Significant differences between H4 and NIL were assessed using Student’s *t*-test; * *P* < 0.05, ** *P* < 0.01. Both x- and y-axes are shown as log2 scale

The miR399 family is involved in phosphate uptake in various plant species [30, 43, 44]. Our results mentioned above suggest that the expression of miR399s family’s expression level was affected by external sucrose. In addition, we observed that phosphate homeostasis-related genes were differentially expressed between H4 and NIL (Fig. 5). In contrast to the expression levels of miR399s, *PHO2* (*GRMZM2G464572* and *GRMZM2G381709*), a miR399 target gene, was more highly expressed in leaves at V2, V3, and V4 stages, while elevated expression was also observed in SAM at V2 and V3 stages in NIL (Fig. 5a-c). Thus, miR399s may regulate *PHO2* at least partially by cleaving this gene during V2 and V3 stages in response to LD conditions. One of six phosphate transporter (PHT) genes, PHT4;1, was differentially expressed between H4 and NIL in leaves and SAM during V2 and V3 stages (Fig. 5d-i). These data imply that miR399-mediated regulation of target genes and essential phosphate transporters during V2 and V3 stages may be affected by LD conditions.

### Inhibition of miR399 expression by external sucrose in *A. thaliana*

**Fig. 6:**
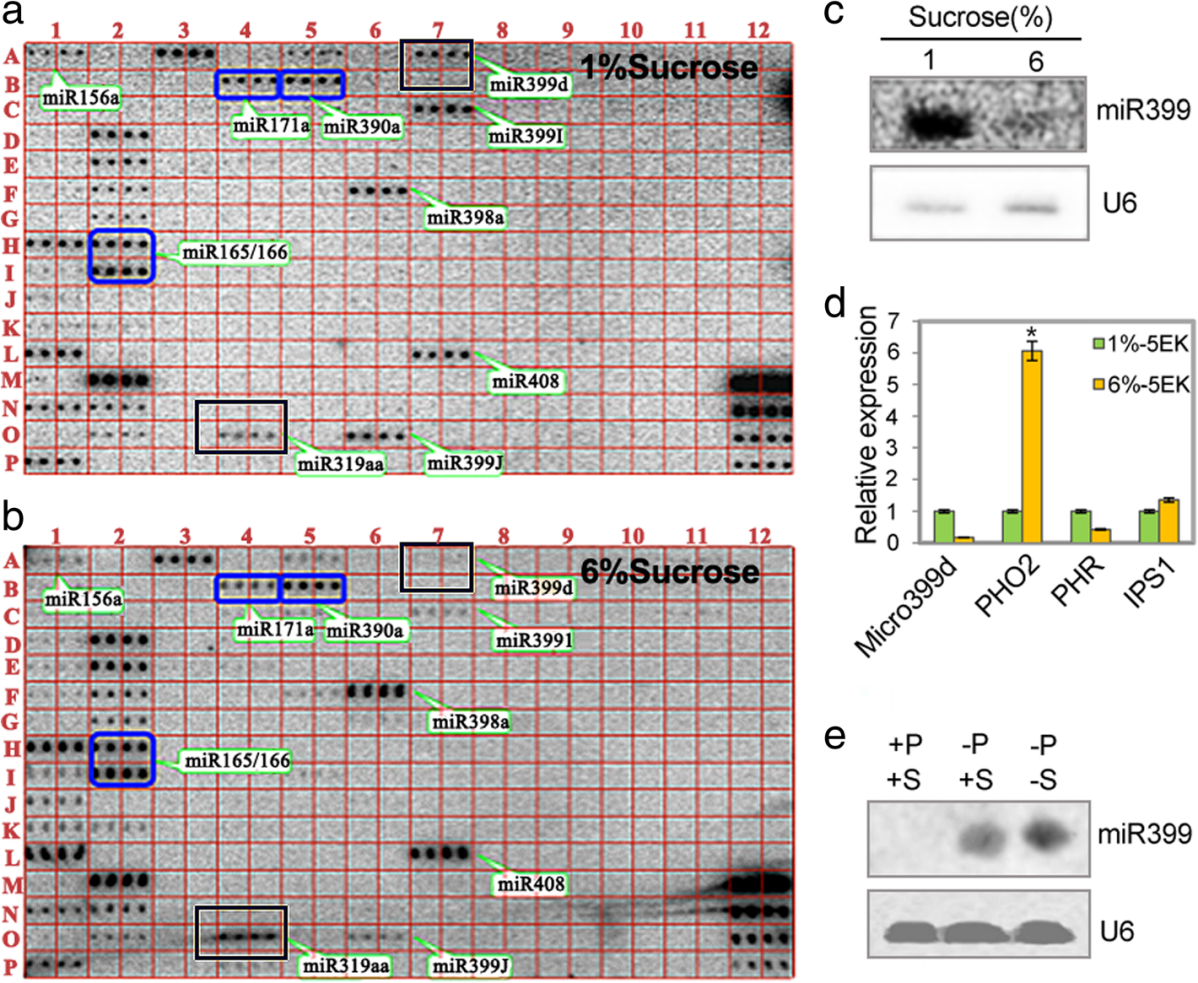
Identification of sucrose-regulated miRNAs from *A. thaliana* subjected to external sucrose supplementation. **a-b** Identification of sucrose-related miRNAs by miRNA array analysis. Total RNAs (100 μg) isolated from 5-week old *A. thaliana* wild-type plants grown on MS medium supplemented with 1% (**a**) and 6% (**b**) sucrose were used for miRNA detection on a 15% PAGE gel. (**c**) Northern blot validation of miR399s in *A. thaliana*. **d** Expression levels of phosphate-responsive genes in 5-week old *A. thaliana* plants subjected to external sucrose supplementation as assessed by northern blotting. **e** Relative expression levels of miR399s in response to external sucrose supplementation under phosphate starvation conditions

To validate the expression of the miR399s family under S+ condition, qRT-PCR was carried out in *A. thaliana* subjected to a supplement of 6% sucrose. Plants grown under the same conditions without any additional sucrose served as controls. As shown in Fig. 6a and b, miRNA399 expression levels were approximately twofold lower in the 6% sucrose-supplemented plants than in S− plants at the 5-week stage (Fig. 6c and d). In addition, the expressions of miRNA399s as revealed by northern blotting, were in plants subjected to phosphate starvation in S+ compared with S−, (Fig. 6e). These results indicated that S+ inhibited miR399 expression during the latter stages of *A. thaliana* development*.* In addition, *A. thaliana* plants grown with 6% sucrose (S+, P+) exhibited later flowering than plants treated with 1% sucrose (S−, P+, the normal growth concentration; Additional file 6). Three weeks after germination, 75% of (S−, P+)-treated plants were flowering, whereas only 16% of (S+, P+) treated plants were flowering or close to flowering (Additional file 6). This result indicated that external sucrose supplements can inhibit the miR399 expression and delays flowering in *A. thaliana*.

## Discussion

### Relationships among miR399s, photosynthesis, and sugars/sucrose in plants

An association between miR399s and photosynthesis has been frequently reported, which indicates that photosynthesis and sugars are related to the P signaling pathway. Stem girdling, a method commonly used to block the transfer of sugar or photosynthates to roots, induces the accumulation of *PSI* gene transcripts in white lupin (*Lupinus albus*). A similar phenomenon has also been observed in response to sucrose, which implies that sugar, sucrose, and photosynthesis play critical roles in plant response to P deficiency [45]. In addition, microarray expression analyses have revealed that the *pho3* mutant exhibits low expression level of the *SUC2* gene, which encodes a sucrose transporter [46, 47]. This result suggests that sugar sensing is tightly related to Pi starvation.

Several regulatory miRNAs have been implicated in maintaining the homeostasis of nutrients, such as phosphate, copper, and salt [48–50]. The miR399 family can be translocated between root and shoots, and P responses in roots may be determined by miR399 translocation in phloem and P levels in shoots [31, 32, 51]. Significantly, sucrose loading and unloading also indirectly influence miR399 responses and P accumulation because of their similar translocation in the phloem [52]. In our study, sucrose accumulated at greater levels in the NIL at the V2 and V3 stages, whereas miR399 expression was lower (Fig. 1d and Additional file 5), thus suggesting a predicted negative correlation between miR399s and internal sucrose in maize.

### Flowering-responsive miRNAs in maize

Changes in photoperiod affect maize output by controlling the phase transition from vegetative to reproductive growth, but photoperiod-regulated miRNAs have not yet been identified in this species under LD condition. In this study, small-RNA-Seq was carried out using leaves and SAM from photoperiod-sensitive and insensitive maize inbred lines grown under LD conditions.

**Fig. 7:**
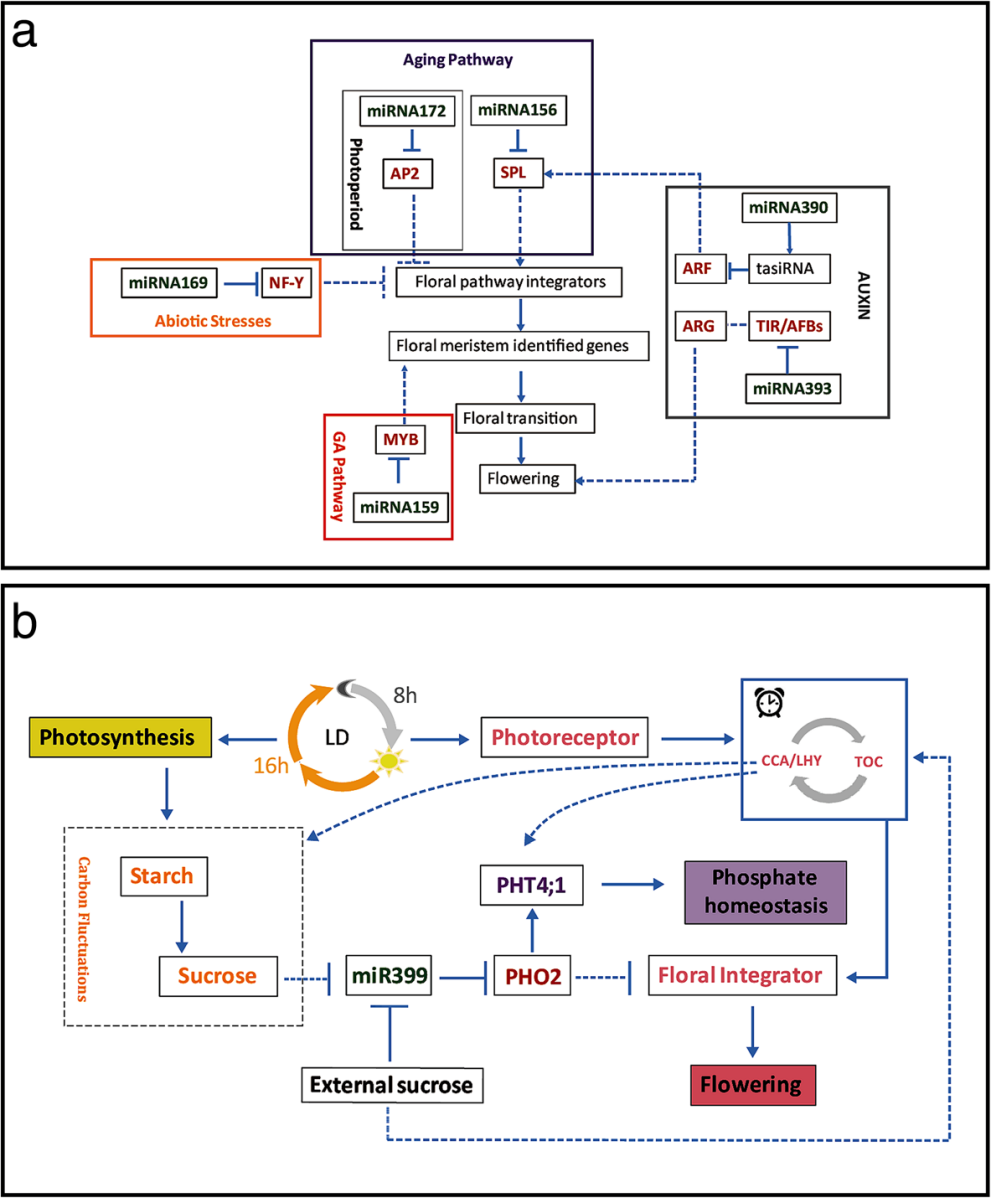
A proposed schematic model of mechanisms involving miRNAs and their target genes under long-day conditions and external stress. **a** Several miRNAs with different expression patterns may be indirectly involved in the plant floral transition by regulating target gene expression levels. The miR156 family, is the main regulator of 11 of the 17 SPL transcription factors. Rising SPL levels positively regulate miR172 expression, resulting in the suppression of the AP2-type floral repressors. The miR159 families regulate MYB transcription factors, respectively and the expression levels rise in response to increased GA signaling. The miR390 family regulates flowering indirectly by promoting the production of tasiRNAs from the TAS3 locus, which negatively regulates the ARF3/4 transcription factors. Additionally, miR393 regulates flowering through controlling over the expression of auxin-responsive genes. The stress-induced miR169-NF-Y module regulates flowering time by inhibiting the floral integrators genes, respectively. **b** Possible regulatory mechanism involving the circadian clock, phosphate homeostasis, and sugar metabolism in plant mediated by miR399-PHO model. Dotted line represents weak interaction in comparison with a solid arrow. Arrows represent activation; line with a bar represents repression. MiRNAs are shown in bold green font and their respective targets are shown in red. Abbreviations: AP2, APETALA 2-TYPE PROTEIN; SPL, SQUAMOSA PROMOTER-BINDING PROTEIN-LIKE; NF-Y, NUCLEAR FACTOR Y; TIR1, TRANSPORT INHIBITOR RESPONSE 1; AFB1 (AUXIN SIGNALING F BOX PROTEIN 1); ARGs, auxin-responsive genes; ARF, AUXIN RESPONSE FACTOR; LD, long day; PHO2, PHOSPHATE 2; GI, GIGANTEA; TOC1, TIMING OF CAB EXPRESSION 1; CCA1, CIRCADIAN CLOCK-ASSOCIATED 1; LHY, LATE ELONGATED HYPOCOTYL(LHY)

Numerous miRNAs, such as miR156, miR159, miR172, and miR169, displayed different expression levels between these two genotypes (Additional file 5). The regulation of these miRNAs during flowering time has been reported in many species, but only miR156 and miR172 are the only conserved miRNAs regulated by LD photoperiod [53–59]. MiR156 and miR172 target genes encode SPL transcription factors and AP2-like family genes, respectively. Together with their targets, these two miRNAs form a network that regulates flowering by controlling downstream genes such as *AP1*, *LFY*, *FUL*, and *FT* [26, 44, 59–62]. These flowering-related genes are also regulated by other miRNAs that are involved in controlling flowering time [53, 57]. In addition, flowering-regulated miRNAs are also controlled by other factors, such as abiotic stress, and hormones including gibberellin and auxin (Fig. 7a).

### MicroRNA 399 as a potential integrator of photo-response, phosphate homeostasis, and sucrose signaling under LD condition

According to Fuji et al. (2005) [48], miR399s are associated with phosphate starvation responses in *A. thaliana*. This miRNA family has been found to be significantly induced under P deficiency, and its target gene *AtUBC24*/*PHO2* has been verified to encode ubiquitin E2-conjugating enzyme [29]. In addition to their involvement in the maintenance of phosphate homeostasis, miR399 and its target gene *PHO2* regulate temperature-responsive flowering in *A. thaliana* [63]. *PHO2* loss of function and miR399b overexpression both cause early flowering by increasing *TWIN SISTER OF FT* expression under LD conditions. In addition, miR399-mediated *PHO2* cleavage may regulate photoperiodic flowering independent of *CO* [63]. In our study, the *PHO2* expression was significantly increased during the vegetative to reproductive transition (Fig. 5), which was the opposite trend to that of miR399 expression. In addition, NIL plants exhibited delayed flowering accompanied by the decreased expression of miR399s and high internal sucrose levels. This suggested that high sucrose level might regulate flowering time in part by inhibiting miR399 expression, thereby leading to later flowering (Fig. 7b). Nevertheless, whether the target gene *PHO2* precisely regulates the expression of the downstream floral genes (such as *FT*) during sucrose responses in maize is still unknown.

Together, miR399 should play an important role in phosphate signaling and the flowering response pathway. In addition, only one gene encoding phosphate transporters was differentially expressed between H4 and NIL, *PHT4;1*. This gene’s expression level was most significantly differences during the V2 and V3 stages (Fig. 5). In an earlier study, recombinant CCA1 protein controlled the circadian expression of *PHT4;1* by binding to the gene’s promoter region, which suggested that the CCA1 protein directly regulates the *PHT4;1* gene at the transcriptional level [64]. Normal circadian control of the degradation rate of starch by *CCA1/LHY* has also been reported and this regulation is essential for preventing growth penalties and sucrose starvation at night [65–67]. According to our qRT-PCR analysis, the *ZmCCA* expression level was elevated in NIL, a maize line that accumulated more sucrose than H4 (Fig. 1d and Additional file 7); in contrast, the expressions of miR399s were lower in NIL at the development transition stage. We concluded that *ZmCCA*-mediated sucrose accumulation may inhibit miR399 expression, but promote the expression of the target gene *PHO2* and phosphate transporter genes to regulate phosphate homeostasis (Figs. 4 and 5). However, further experimental evidence is needed to precisely define the roles of *ZmCCAs*, miR399s, and sucrose in the maize LD-photoperiodic pathway (Additional file 7).

## Conclusions

In this study, we used the photoperiod-sensitive NIL, which has a higher sucrose accumulation and a delayed flowering phenotype compared with the photoperiod-insensitive line H4, to explore the relationships among LD conditions, miR399s, and sucrose. External sucrose supplementation in maize and *A. thaliana* demonstrated that high external sucrose can inhibit the expression of miR399s. In addition, the higher sucrose accumulation in the NIL observed in vivo, accompanied by the higher miR399 expression may provide new insights on the integration of sucrose and miR399 family levels with flowering time regulation under LD conditions. Finally, our findings help establish a novel link between LD-photoperiod and carbon metabolism in plants.

## Additional files

Additional file 1: Primers used in the quantitative real-time RT-PCR analysis. (XLSX 10 kb)

Additional file 2: MiRNA families identified in the leaves and SAM of the maize H4 and NIL inbred lines. (XLSX 12 kb)

Additional file 3: Predicted targets of known and novel miRNAs. (XLSX 372 kb)

Additional file 4: Functional enrichment analyses of miRNA targets based on gene ontology (GO). (XLSX 27 kb)

Additional file 5: Differentially expressed miRNAs in leaves and SAM between H4 and NIL. (XLSX 106 kb)

Additional file 6: Phenotypes of *A. thaliana* grown MS medium supplemented with high sucrose at different developmental phases. (**a**) Phenotypes of 2-week-old plants under high sucrose conditions. (**b**) Percentages of flowering plants at different developmental phases. (TIF 805 kb)

Additional file 7: Expression levels of CCA genes in the maize H4 and NIL lines. (TIF 167 kb)

## Abbreviations

CO: Constans
FT: Flowering locus t
GI: Gigantea
GO: Gene ontology
LD: Long day
miRNA: MicroRNA
qRT-PCR: Quantitative real-time PCR
SAM: Shoot apical meristem
SOC1: Supressor of overexpression of constans1
SPL: Squamosa promoter binding protein-like
ZCN1: *Zea mays* CENTRORADIALIS1
