# Supplementary figures and images for: MicroRNA 399 as a potential integrator of photo-response, phosphate homeostasis, and sucrose signaling under long day condition
Source: BMC Plant Biol. 2018 Nov 21;18:290. doi: 10.1186/s12870-018-1460-9 (PMC6249786; doi:10.1186/s12870-018-1460-9)

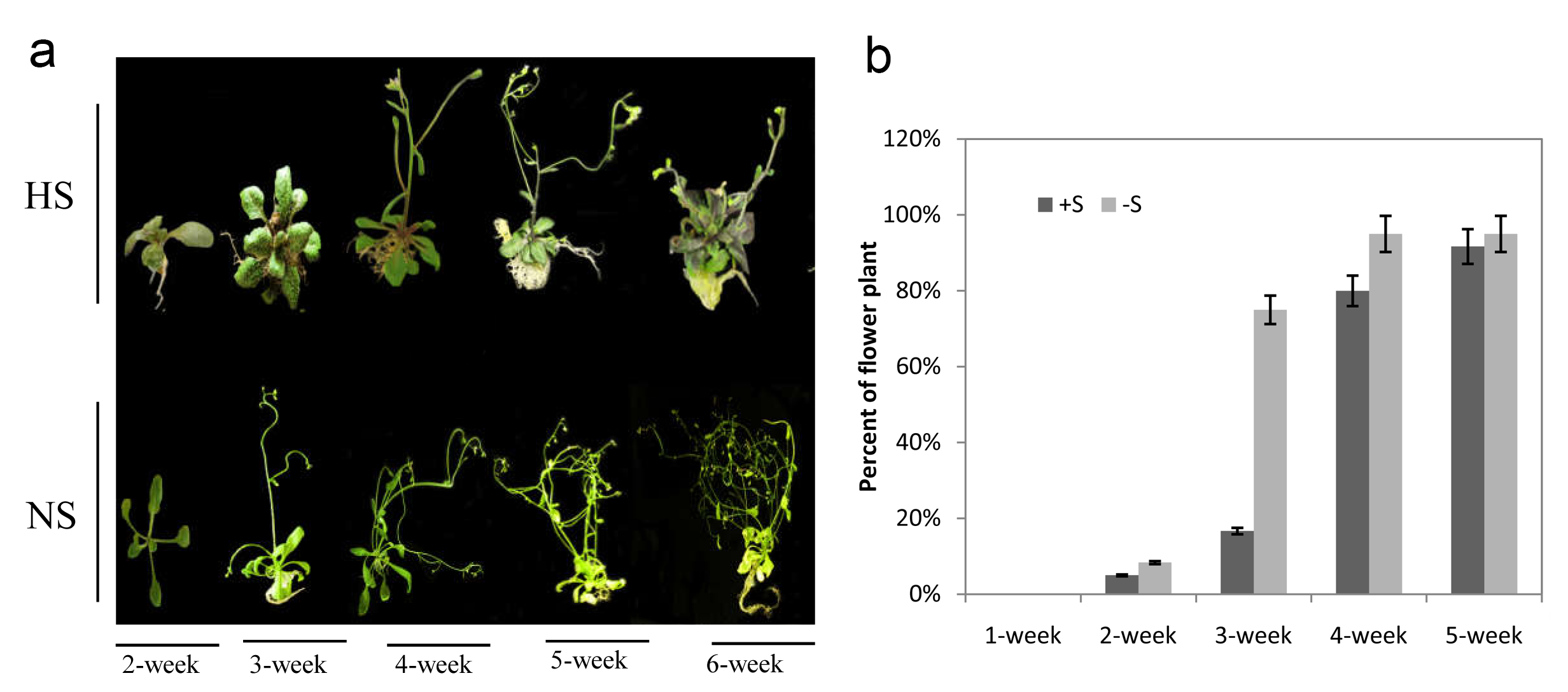

Supplement: Supplementary file 6 — Phenotypes of A. thaliana grown MS medium supplemented with high sucrose at different developmental phases. (a) Phenotypes of 2-week-old plants under high sucrose conditions. (b) Percentages of flowering plants at different developmental phases. (TIF 805 kb) [file 12870_2018_1460_MOESM6_ESM.tif]

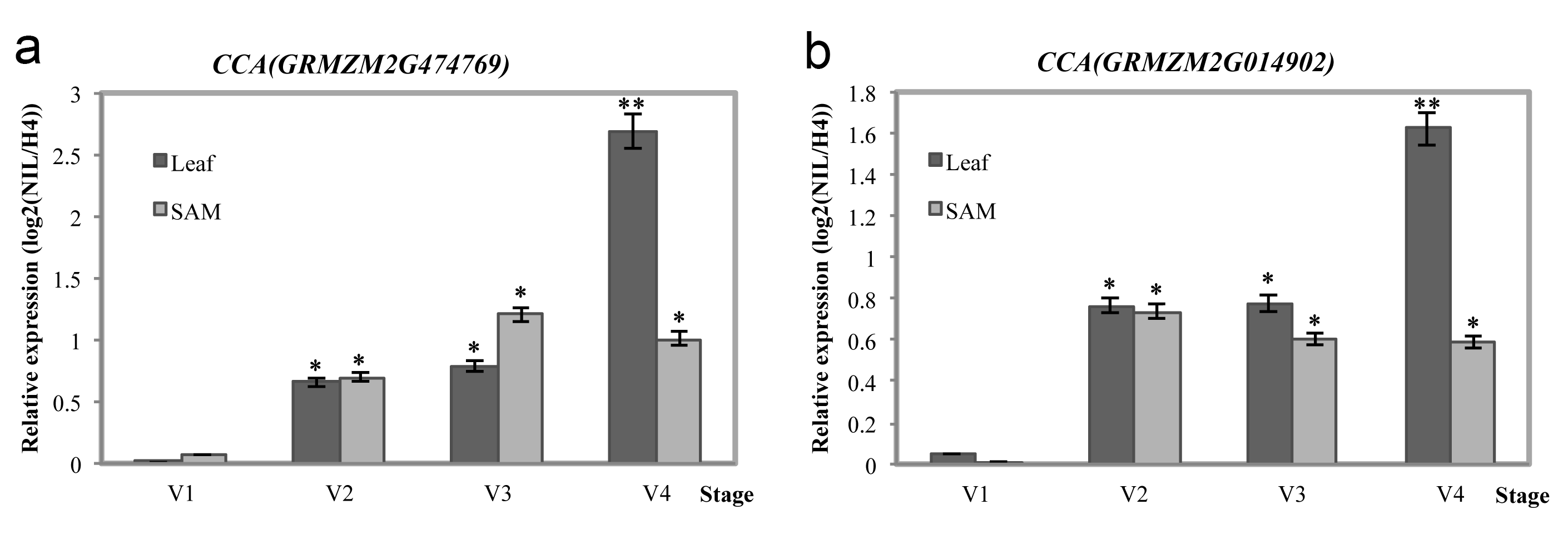

Supplement: Supplementary file 7 — Expression levels of CCA genes in the maize H4 and NIL lines. (TIF 167 kb) [file 12870_2018_1460_MOESM7_ESM.tif]
